# Efficacy and safety of Chinese patent medicine injection for COVID-19: A protocol for systematic review and meta-analysis

**DOI:** 10.1097/MD.0000000000020706

**Published:** 2020-06-19

**Authors:** Meiqi Lu, Zhen Lu, Ting Zhang, Wei Wang, Ye Xue, Zhiqun Cao

**Affiliations:** a The First Clinical College, Shandong University of Traditional Chinese Medicine; b Department of Pharmacy, Shandong Rehabilitation Hospital; c The Affiliated Hospital of Shandong University of Traditional Chinese Medicine; d Department of Internal Medicine of Traditional Chinese Medicine, Shandong University of Traditional Chinese Medicine, Jinan, Shandong Province, China.

**Keywords:** Chinese patent medicine injection, COVID-19, protocol, systematic review

## Abstract

**Background:**

Corona Virus Disease 2019 (COVID-19) has caused a worldwide epidemic since its discovery. The outbreak of virus infection has aroused great concern of the World Health Organization (WHO). COVID-19 is highly infectious and has a high infection rate. So far, no specific drug has been found to cure it. China as one of the first countries attacked by epidemic has shown outstanding in fighting against the COVID-19. The contribution of traditional Chinese medicine can not be ignored. As a kind of representative of traditional Chinese medicine, the Chinese patent medicine injection has significant effect in reducing the clinical symptoms of patients and preventing the deterioration of the disease. However, there is no systematic review of its efficacy and safety. The purpose of this study is to evaluate the efficacy and safety of Chinese patent medicine injection in the treatment of COVID-19.

**Methods:**

All randomized controlled trials of Chinese patent medicine injection for COVID-19 will be included. The following electronic databases will be searched: PubMed, Web of Science, the Cochrane Library, EMBASE, China National Knowledge Infrastructure, Wanfang Database, Chinese Scientific Journal Database, Chinese Biomedical Literature database and some clinical trial registration websites. Two researchers will independently screen titles, abstracts, full texts, and extract data, then assess the bias risk of each study. We will conduct meta-analyses to assess all the available evidence of the efficacy and safety.

**Results:**

Systematic review of current evidence will be provided from the indexes of efficacy and safety.

**Conclusion:**

Evidence regarding the efficacy and safety of Chinese patent medicine injection in the treatment of COVID-19 will be provided to clinicians.

PROSPERO registration number: CRD42020182725

## 1 Introduction

Corona Virus Disease 2019 (COVID-19) is an acute respiratory syndrome caused by novel coronavirus “2019-nCoV”, which has a large-scale epidemic^[1,2]^ and has aroused great concern in global public health.^[3]^ The epidemic of COVID-19 has been controlled in China, but the virus is still spreading in Europe and North America.^[4]^ Spain is currently one of the countries most affected by COVID-19, with 210,773 confirmed cases as of April 28, 2020.^[5]^ Similarly, Italys situation is no better.^[6]^ The main clinical manifestations of COVID-19 patients were fever, cough and fatigue, and some patients also had nasal congestion, runny nose, and diarrhea.^[7]^ COVID-19 can spread from person to person,^[8,9]^ and is highly infectious, with a variety of transmission modes (droplets, close contact, faecal, etc.).^[10,11]^ The population is generally susceptible to infection. Western medicine mainly adopts antiviral, oxygen therapy, nutritional support and other standard supportive treatment,^[12,13]^ but there is still a lack of specific drugs.^[14]^ Vaccine development is progressing rapidly, but the first wave of the pandemic may not be contained.^[15]^

In traditional Chinese medicine, COVID-19 is classified as “epidemic disease”, which is caused by the feeling of the Qi of disease. Traditional Chinese medicine has a long history in the treatment of epidemic diseases,^[16]^ and has accumulated rich clinical experience. At present, many authoritative experts of traditional Chinese medicine believe that COVID-19 may be related to dampness, heat, toxin, blood stasis, Qi deficiency, turbid toxin and turbid dampness.^[17,18]^ In the fight against COVID-19 epidemic, Chinese medicine played a significant role. The specific manifestations are as follows: the combination of traditional Chinese medicine and Western medicine can reduce the clinical symptoms of common patients, shorten the course of treatment, and promote healing; for severe and critical patients, it can reduce the lung exudation, control the inflammatory overreaction, and prevent the deterioration of the disease; for patients in the recovery period, it can remove the residual pathogens, support the healthy qi, and promote the recovery of the body.^[18]^

As a new and unprecedented infectious disease, COVID-19 has become a global concern of the World Health Organization (WHO) because of its rapid spread around the world.^[19]^ The early intervention of traditional Chinese medicine and the combination of traditional Chinese and Western medicine are important means to improve the cure rate of the COVID-19 and reduce the mortality.^[20]^ Therefore, it is necessary for us to evaluate the efficacy and safety of Chinese patent medicine injection in the treatment of COVID-19 based on the existing evidence. In order to better guide the clinical application of Chinese patent medicine injection for COVID-19 and provide better treatment options. It is urgently needed to accomplish this review.

## 2 Objective

This work aims to evaluate the current evidence for the efficacy and safety of Chinese patent medicine injection in treating COVID-19, and provide reliable evidence-based medical evidence for the clinical treatment of COVID-19.

## 3 Methods

This protocol has been registered in the International Prospective Systematic Registration Review (PROSPERO) and the registration number is CRD42020182725.

This study has followed the guidelines of Preferred Reporting Items for Systematic Review and Meta-Analysis Protocols (PRISMA-P).^[21]^

### 3.1 Inclusion criteria

#### 3.1.1 Types of studies

This study includes all relevant randomized controlled trials (RCTs) of Chinese patent medicine injection for treating COVID-19 published in Chinese or English, regardless of allocation hidden or blinded.

#### 3.1.2 Types of population

The population included patients diagnosed with COVID-19, regardless of gender, age, race, nationality, and other characteristics.

#### 3.1.3 Interventions and comparisons

The patients in treatment group must have been treated with a kind of Chinese patent medicine injection (including Xiyanping injection, Xuebijing injection, Reduning injection, Tanreqing injection, Xingnaojing injection, Shenfu injection, Shengmai injection, Shenmai injection), whether combined with other pharmacotherapies or applied separately. The treatment of control group was commonly used symptomatic medication such as antiviral, anti-infective, nutritional support drugs, or no treatment was given. Once treated with one of Chinese patent medicine injections, the trial will be excluded.

#### 3.1.4 Outcomes.

The primary outcomes of our interest are as follows:

1. clinical effective rate (rate of positive to negative for nucleic acid test),
2. disappearance rate of main symptoms (including fever, fatigue, cough,etc.),
3. occurrence rate of light or common type to severe form,
4. mortality rate.

The secondary outcomes of our interest are as follows:

1. improvement of secondary symptoms (chest pain, difficulty breathing, muscle ache, nausea, vomiting, diarrhea, etc),
2. chest computed tomography (CT) imaging improvement,
3. peripheral blood index detection like C-reactive protein (CRP), procalcitonin (PCT), white blood cell count (WBC), lymphocyte count (LYM),
4. safety index, incidence of adverse events.

### 3.2 Exclusion criteria

The exclusion criteria were as follows:

1. non-randomized controlled trial, and self-control,
2. case report,
3. experience summary,
4. animal experiment research,
5. systematic review, and meta-analysis.

### 3.3 Database and search strategy

We will use the computer to search the following electronic bibliographic databases: PubMed, Web of Science, the Cochrane Controlled Trials Central Register System (CENTRAL) Cochrane Library, EMBASE, China National Knowledge Infrastructure (CNKI), Wanfang Database, Chinese Scientific Journal Database (VIP database), and Chinese Biomedical Literature database (CBM). Limited the search time from the establishment of databases to May 2020. We will apply a combination of MeSH terms and free-text to search and adjust the search strategy according to the characteristics of each database. Pubmeds detailed search strategy is shown in Table 1. In addition, the ongoing trials registered in the Chinese Clinical Trial Registry Website (www.chictr.org.cn), American Clinical Trials Registry Website (clinicaltrials.gov), World Health Organizations International Clinical Trials Registry Platform (WHO ICTRP) will also be searched.

**Table 1:**
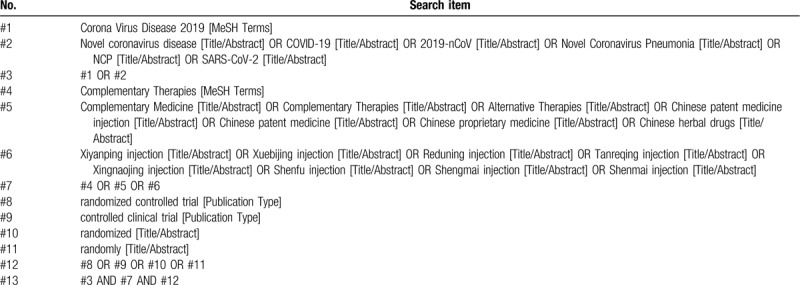
Details of the search strategy of PubMed.

### 3.4 Data collection and analysis

#### 3.4.1 Identification and selection of studies

**Figure 1:**
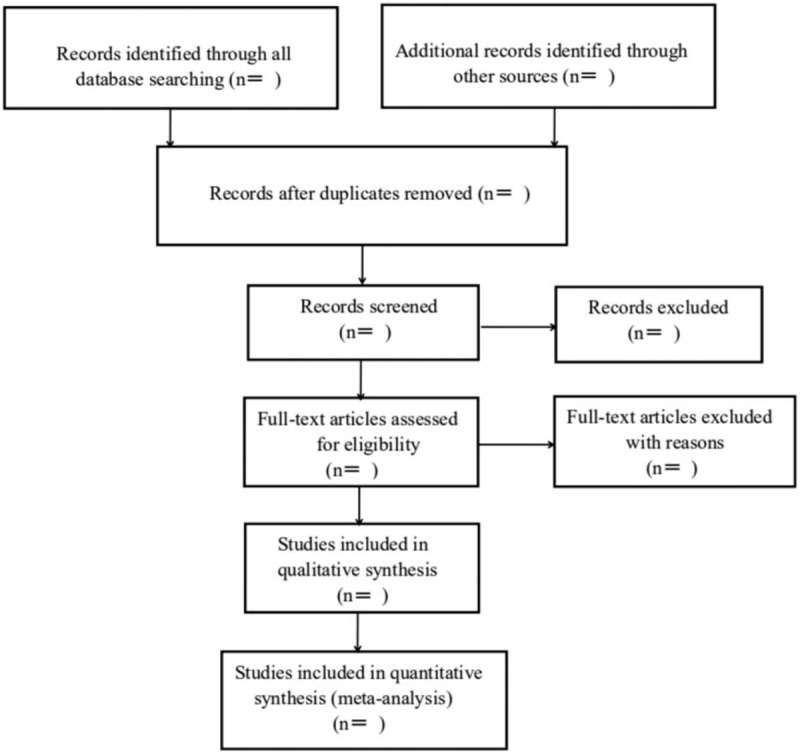
Flow diagram of literature retrieval. A flow diagram of selection process for literature retrieval.

Firstly, we will use EndNoteX7 software to find the repeated title information. When the retrieval is complete, repeated references will be deleted. The 2 researchers (ML and ZL) then independently screen the titles and abstracts to rule out apparently unrelated studies. Subsequently, the literature after preliminary screening will be inspected in full text to determine whether to be included. The details of selection process are shown in Figure 1. In this process of extracting, the data will be filled into the pre-established tables. If differences are encountered, they will be resolved by discussion or by seeking opinions from a third party (ZC).

#### 3.4.2 Data extraction and management

The 2 reviewers (ML and TZ) will independently select the literature and extract the data according to the established retrieval strategy, and discuss or consult the third reviewer (WW) to make a decision in case of disagreement. During the selection and identification of studies, the 2 reviewers (ML and TZ) first read the title and abstract of each literature, excluding unrelated studies. The second step is to read the full text of the literature initially identified for inclusion. During the data extraction, the Microsoft Excel data extraction form will be used to extract the data from the literature included. We will attempt to extract the following data information from each study: first author, title, country/region, year of publication, study design, sample size, intervention approach, outcome indicators, adverse events. If the required data is missing, we will try to contact the literature authors by e-mail or phone to ensure the accuracy of the relevant information.

#### 3.4.3 Measure of treatment effect

Two reviewers (ML and ZL) will analysis the treatment effect by Review Manager 5.3. Statistics for the analysis of efficacy indicators will use odds ratio (OR), continuous variables will use weighted mean difference (WMD), and give 95% confidence interval (CI).

#### 3.4.4 Risk of bias assessment

Risk of bias in the included studies will be assessed by the Cochrane Risk of Bias Tool^[22]^ according to the Cochrane Handbook 5.1.0 for Systematic Reviews of Interventions, which consists of 7 items of bias relevant to the quality of RCTs. The criteria to be assessed include the following domains: random sequence generation (selection bias), allocation concealment (selection bias), blinding of participants and personnel (performance bias), blinding of outcome assessment (detection bias), incomplete outcome data (attrition bias), selective reporting (reporting bias), and other bias. An assessment of risk of bias will be made for the included studies based on the following 3 levels: “low risk of bias,” “unclear risk of bias,” “high risk of bias.” Such an evaluation process will be independently performed by 2 researchers (ML and YX), and when differences arise, a third person (WW) will be required to participate in the discussion to determine the risk of bias.

#### 3.4.5 Data analysis

Using Review Manager 5.3 software provided by Cochrane Collaboration Network, meta-analyses will be carried out for the included researches. When *P* > .05, we generally determined that there is no heterogeneity among the studies, fixed-effects model is selected for analysis, otherwise random-effects model is selected. In addition, we also use *I*^2^ for quantitative analysis of heterogeneity,^[23]^ it is generally considered that *I*^2^ > 50% indicates the existence of substantial heterogeneity. When there is homogeneity among the studies, the fixed-effects model analysis is used; when there is heterogeneity between the studies, the random-effects model analysis is used. If we find a significant level of heterogeneity, a descriptive analysis will be performed instead.

#### 3.4.6 Subgroup analysis

If the included evidence is rich, we will conduct a subgroup analysis of the factors that influence the outcome, such as: disease types of COVID-19 and whether accompanied by underlying disease.

#### 3.4.7 Sensitivity analysis

Considering that the diversity of included studies will lead to a certain degree of heterogeneity and inconsistency, we will conduct a sensitivity analysis. This process will be carried out by eliminating each included study. If the heterogeneity does not change after excluding each literature, we think our conclusion is stable; otherwise, if the heterogeneity changes, the excluded literature may be the source of heterogeneity.

#### 3.4.8 Assessment of publication bias and quality of the evidence

Where a sufficient number of trials per comparison are available in a result of meta-analysis, the funnel plot will be drawn to analyze the publication bias of the included studies. If the included studies were distributed symmetrically on both sides of the equivalent line, the possibility of publication bias will be small. The evidence quality of the study will be assessed by the Grading of Recommendations Assessment, Development and Evaluation (GRADE) framework.^[24]^ The 4 levels of the GRADE for the quality of evidence are very low quality, low quality, moderate quality, and high quality.

## 4 Discussion

COVID-19, as a global health hotspot that continues to ferment, has attracted wide attention for its therapeutic methods and drugs. Since the outbreak of the epidemic, Chinese medicine has been widely used in the treatment of COVID-19 and has played a prominent role in disease prevention and control. The purpose of this study was to evaluate the efficacy and safety of Chinese patent medicine injection in the treatment of COVID-19 and to explore the role of Chinese medicine in the treatment of COVID-19. At present, relevant studies have reported that integrated Chinese and Western medicine treatment can safely and effectively reduce the clinical symptoms of patients, reduce the case fatality rate and the conversion rate of critically ill patients.^[25,26]^ Therefore, traditional Chinese medicine is a potential treatment method for COVID-19. This study will systematically evaluate the efficacy and safety of Chinese patent medicine injection for COVID-19 based on available evidence. The presentation of these results will provide better options and more compelling evidence for clinical treatment. Considering that there may be many studies that have not yet been published, more high-quality clinical evidence will need to be updated to ensure the integrity of the evidence in this study.

## Author contributions

**Conceptualization:** Meiqi Lu, Zhiqun Cao.

**Data curation:** Meiqi Lu, Zhen Lu, Ting Zhang, Wei Wang.

**Formal analysis:** Meiqi Lu, Zhen Lu

**Funding acquisition:** Zhiqun Cao.

**Methodology:** Meiqi Lu, Ting Zhang, Zhen Lu.

**Resources:** Ye Xue.

**Software:** Meiqi Lu, Ye Xue, Ting Zhang.

**Supervision:** Wei Wang.

**Writing – original draft:** Meiqi Lu.

**Writing – review & editing:** Meiqi Lu, Zhiqun Cao
